# Systems Analysis and Improvement Approach to optimize the pediatric and adolescent HIV Cascade (SAIA-PEDS): a pilot study

**DOI:** 10.1186/s43058-022-00272-8

**Published:** 2022-05-10

**Authors:** Anjuli D. Wagner, Orvalho Augusto, Irene N. Njuguna, Douglas Gaitho, Nancy Mburu, Geoffrey Oluoch, Naziat Carimo, Peter Mwaura, Peter Cherutich, Laura Oyiengo, Sarah Gimbel, Grace C. John-Stewart, Ruth Nduati, Kenneth Sherr

**Affiliations:** 1 Department of Global Health, University of Washington, Box 359931, Seattle, WA 98104 USA; 2 Universidade Aduardo Mondlane, Maputo, Mozambique; 3 Research & Programs, Kenyatta National Hospital, Nairobi, Kenya; 4 Network of AIDS Researchers in Eastern and Southern Africa, Nairobi, Kenya; 5 Ministry of Health, Nairobi, Kenya; 6 National AIDS & STI Control Programme, Ministry of Health, Nairobi, Kenya; 7 Department of Child, Family and Population Health Nursing, University of Washington, Seattle, USA; 8 Department of Epidemiology, University of Washington, Seattle, USA; 9 Deptartment of Pediatrics, University of Washington and Department of Medicine, University of Washington, Seattle, USA

**Keywords:** Pediatric, Adolescent, HIV, HIV testing, HIV treatment, Virologic testing, Virologic suppression, Health systems, Implementation science, Systems engineering, Cascade analysis, Flow mapping, Continuous quality improvement

## Abstract

**Introduction:**

Children and adolescents lag behind adults in achieving UNAIDS 95-95-95 targets for HIV testing, treatment, and viral suppression. The Systems Analysis and Improvement Approach (SAIA) is a multi-component implementation strategy previously shown to improve the HIV care cascade for pregnant women and infants. SAIA merits adaptation and testing to reduce gaps in the pediatric and adolescent HIV cascade.

**Methods:**

We adapted the SAIA strategy components to be applicable to the pediatric and adolescent HIV care cascade (SAIA-PEDS) in Nairobi and western Kenya. We tested whether this SAIA-PEDS strategy improved HIV testing, linkage to care, antiretroviral treatment (ART), viral load (VL) testing, and viral load suppression for children and adolescents ages 0–24 years at 5 facilities. We conducted a pre-post analysis with 6 months pre- and 6 months post-implementation strategy (coupled with an interrupted time series sensitivity analysis) using abstracted routine program data to determine changes attributable to SAIA-PEDS.

**Results:**

Baseline levels of HIV testing and care cascade indicators were heterogeneous between facilities. Per facility, the monthly average number of children/adolescents attending outpatient and inpatient services eligible for HIV testing was 842; on average, 253 received HIV testing services, 6 tested positive, 6 were linked to care, and 5 initiated ART. Among those on treatment at the facility, an average of 15 had a VL sample taken and 13 had suppressed VL results returned.

Following the SAIA-PEDS training and mentorship, there was no substantial or significant change in the ratio of HIV testing (RR: 0.803 [95% CI: 0.420, 1.532]) and linkage to care (RR: 0.831 [95% CI: 0.546, 1.266]). The ratio of ART initiation increased substantially and trended towards significance (RR: 1.412 [95% CI: 0.999, 1.996]). There were significant and substantial improvements in the ratio of VL tests ordered (RR: 1.939 [95% CI: 1.230, 3.055]) but no substantial or significant change in the ratio of VL results suppressed (RR: 0.851 [95% CI: 0.554, 1.306]).

**Conclusions:**

The piloted SAIA-PEDS implementation strategy was associated with increases in health system performance for indicators later in the HIV care cascade, but not for HIV testing and treatment indicators. This strategy merits further rigorous testing for effectiveness and sustainment.

Contributions to the literature

- Fewer implementation strategies address system-level barriers and health system organization in HIV testing and treatment.
- We tested an adapted multi-component, systems-focused implementation strategy in 5 clinics in Kenya to improve the pediatric and adolescent HIV cascade. This strategy was previously shown to improve HIV program performance for pregnant people living with HIV.
- We found that the implementation strategy was associated with improvements in some, but not all, steps in HIV testing and care. The most meaningful improvements were in viral load testing.
- These findings provide evidence that this adapted implementation strategy is feasible and potentially effective in a low-resource settings and merits broader testing.

## Introduction

Children and adolescents living with HIV lag behind adults in reaching the UNAIDS 95-95-95 goals for HIV testing, HIV treatment, and viral load (VL) suppression [1]. While World Health Organization guidelines recommend universal HIV testing for children and adolescents seeking outpatient and inpatient care, as well as immediate test-and-treat strategies for all ages, in 2020, just 53% of children living with HIV globally were receiving life-saving antiretroviral therapy (ART), compared to 68% of adults [1]. ART adherence and virologic suppression require continued adherence to often unpalatable pediatric formulations of medications, and regular visits to health facilities for monitoring.

Barriers to HIV care at all steps of the cascade occur at the systems- and individual-level. At the individual level, children rely heavily on caregivers, while adolescents have emerging autonomy, both facing challenges navigating health systems and maintaining engagement and adherence in chronic care. At the system-level, health care providers and clients face staffing shortages, stock outs of essential supplies, increasing responsibilities to deliver for large populations, unclear clinic flow, and documentation and tracking systems that allow for gaps in coverage. This combination of individual and systems-level barriers yields sub-optimal service delivery for children and adolescents [2–5]. While many strategies focus on individual barriers, fewer have focused on addressing system-level barriers and health system organization.

The Systems Analysis and Improvement Approach (SAIA) is a multi-component implementation strategy to address health systems organization; SAIA combines three systems engineering [6] tools—flow mapping, cascade analysis [7], and continuous quality improvement—to identify and prioritize gaps in service delivery and identify and test micro-interventions to optimize system performance. SAIA was effective in reducing drop offs in the prevention of mother-to-child transmission of HIV (PMTCT) cascade, specifically in improving ART coverage and infant HIV testing [8]. This flexible implementation strategy has been adapted to different service delivery platforms [9–12]. Pediatric and adolescent HIV care systems share similar cascade steps, cadres of health care workers, and physical space transitions with PMTCT systems.

In this pilot study, we aimed to define the pediatric and adolescent HIV cascade, characterize the cascade in the absence of the implementation strategy, and pilot and measure the effect of the adapted SAIA-PEDS strategy in Kenya. We assessed the impact of SAIA-PEDS on pediatric and adolescent HIV testing, linkage to care, treatment initiation, VL monitoring, and VL suppression.

## Methods

### Study setting

This pre-post pilot was conducted between July 2017 and June 2018 at six government health facilities in Kenya: three in Nairobi County, one in Homa Bay County, one in Kisumu County, and one in Siaya County. The six facilities were purposively selected to represent diversity in size and level of services, with two County Hospitals, two sub-County hospitals, and two health centers. All facilities provided comprehensive HIV testing and care services, with VL samples sent to centralized laboratories for processing. All invited facilities initially agreed to participate, but due to delays in implementation, one facility in Homa Bay was excluded from analyses, leaving a final sample size of five facilities. During the year prior to the introduction of the implementation strategy, there were two nation-wide health care worker strikes and an initial and repeated presidential election; these events have been documented to have negatively impacted service delivery across Kenya [13–15].

### Ethical approval

This study was reviewed and approved by the University of Washington Institutional Review Board, the Kenyatta National Hospital Ethics and Research Committee, and the National Commission for Science, Technology, and Innovation (NACOSTI). Additionally, following ethical approval, the study was reviewed and approved by County and sub-County health offices, and further permission was sought from each facility’s medical superintendent and in-charge prior to facility engagement.

### SAIA implementation strategy

SAIA consists of three systems engineering [6] tools that are utilized in a cyclical approach by frontline health care workers and managers to identify and prioritize gaps in service delivery and test micro-interventions to improve care delivery systems: cascade analysis tool [7], flow mapping, and continuous quality improvement [8, 16, 17].

#### Pediatric/adolescent Cascade analysis tool (PedCAT)

The cascade analysis tool (CAT) [7] is an Excel-based simple simulation model with an optimization function. The CAT is populated by routine program data for a specific facility and automatically quantifies the drop off at each step of the HIV cascade and quantifies the additional number of individuals who would complete all steps of the cascade if each single step were individually optimized. The goal of the CAT is to quantify and prioritize gaps in service delivery and allow frontline health care workers to access and interpret their own data.

**Fig. 1:**
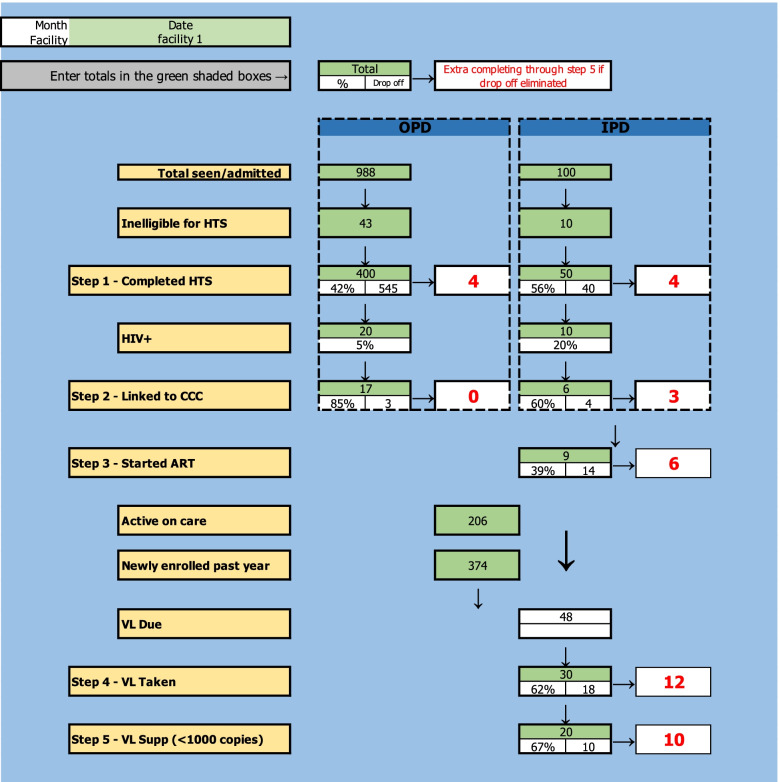
PedCAT component of the SAIA-PEDS implementation strategy with dummy data. The green cells are entered by health care workers using routine program data sources; the white cells are automatically calculated by the Excel sheet. The numbers in red represent the “cascade gain,” the number of individuals who would successfully complete all steps of the cascade if that step, and only that step, was fully optimized. The development and considerations of the PedCAT have been published elsewhere

This tool required adaptation from the original SAIA package to be applicable to the pediatric and adolescent HIV cascade, adapting the original CAT to be the PedCAT. We conducted a physical walk through of each pilot health facility to characterize health information registers, cards, and other data collection and reporting tools; observe patient flow; and ask each operator of the health system to describe what activities were conducted at each step. Following this data mapping activity, an initial tool was created and presented to clinic managers and frontline health care workers to determine whether the service flow modeled in the tool reflected realistic flow patterns, made realistic assumptions, and was sufficiently simple to be useful for routine use; this process was similar to member checking in qualitative research. We conducted several rounds of revisions to the PedCAT before a final tool was agreed upon (Fig. 1).

#### Flow mapping

Flow mapping, also known as value stream mapping or process mapping, consists of frontline health care workers creating a visual map of their health system, drawing the sequential steps taken by clients, data, or samples; the goal of flow mapping is to identify system inefficiencies and bottlenecks and also visualize system reorganization [6, 18].

#### Continuous quality improvement (CQI)

CQI has a large body of effectiveness literature supporting its use in a range of settings [19–21], and there are diverse ways in which CQI is delivered. In this study, we utilized the Model for Improvement and “plan, do, study, act (PDSA)” cycles, in which health care worker teams address the following questions in a group setting: What are we trying to accomplish? How will we know a change is an improvement?, and What change can we make that will result in an improvement? and then Plan the details of a test of a micro-change, Do the micro-change, Study whether the micro-change impacted an identified indicator, and Act to either adapt, adopt, or abandon that micro-change based on the indicator data [22].

#### Intended use of tools

The three SAIA-PEDS tools are intended for combined use in a cyclical way, with flexibility to more or less heavily utilize tools that health care workers find useful or burdensome in a given local setting.

### Training and staffing of implementation strategy

Three study staff members were responsible for training frontline health care workers in the SAIA-PEDS tools, and two of these study staff members were responsible for periodic visits to the facilities to coach and mentor frontline health care workers in the use of the SAIA-PEDS tools. The study staff members received intensive training in PedCAT interpretation, flow mapping, and CQI coaching; prior to study activities, both study team members had experience in clinical care for children and adolescents in Kenya.

Frontline health care workers were trained together in a half-day offsite session; facility in-charges were responsible for selecting and recruiting at least one representative from each of the following service delivery areas: outpatient, inpatient, HIV testing services, HIV care clinic, and laboratory to attend the training. Training covered the basics of PedCAT interpretation, the basics of CQI with a practical exercise in “plan, do, study, act (PDSA),” and included creating a flow map of a facility’s patient flow. Following this half day training, study staff visited each facility for a facility-wide sensitization meeting, which covered the intent of the implementation strategy and allowed all facility staff to ask questions.

### Schedule of follow-up visits and data collection at facilities

The intended schedule for coaching and mentorship visits by study staff to each facility was weekly for the first 1 month, every 2 weeks for the next 2 months, and monthly for the final 3 months. The intended meeting members were the frontline health care workers trained in the initial training, but substitutions could be made by the in-charge due to staff turnover or transfer. During each 1–2-h coaching and mentorship meeting, study staff guided the facility team through reviewing their micro-changes using PDSA cycles, reviewing data that facility staff had collected to inform indicators to evaluate micro-changes. The PedCAT and flow mapping tools were used as needed to identify and prioritize gaps and brainstorm service flow reorganization.

### Data sources and outcome definitions

We considered a range of routine data sources (described in detail elsewhere [7]) with the intent of using easily accessible and accurate data that allowed disaggregation of children (0–9 years), adolescents (10–19 years), and young adults (20–24 years). Ultimately, paper registers and electronic medical records were utilized; two data abstractors per facility were engaged to abstract data from paper registers or electronic medical records, depending on the facility’s data systems. We abstracted anonymous, individual-level, count data aggregated to the calendar day and age band (0–4, 5–9, 10–14, 15–19, and 20–24 years) during data collection. Count data were entered on tablets using Open Data Kit [23]. Daily count data were subsequently aggregated to the month during data cleaning.

Five outcome variables were assessed: HIV testing uptake: # children and adolescents who received HIV testing services (numerator)/# children and adolescents who presented to outpatient or inpatient departments (denominator); Linkage to care: # children and adolescents with new HIV care files (numerator)/# children and adolescents who were reactive in HIV testing (denominator); ART initiation: # children and adolescents starting ART (numerator)/# children and adolescents who were linked to care (denominator); VL monitoring: # children and adolescents with a VL sample collected (numerator)/# children and adolescents due for VL testing (denominator); VL suppression: # children and adolescents with VL < 1000 copies/mL (numerator)/# children and adolescents with VL samples taken (denominator).

All numerator and denominator data were directly abstracted from registers with the exception of the number of children due for a VL sample, which was calculated as a monthly average based on the HIV care guidelines at the time, which indicated six-monthly VL monitoring during the first year of treatment, followed by annual VL monitoring. Of note, the individuals in the numerator and denominator of each outcome were not required to be the same individuals; this was not a longitudinal cohort. As a result, the ratios of numerator to denominator often exceeded one, particularly for indicators where substantial in-migration was common; for example, some facilities had substantial numbers of children diagnosed with HIV at other facilities linking to care at their facility for HIV care services. Conversely, the ratio of numerator to denominator cannot be accurately interpreted as proportions or absolute coverage because some groups of individuals may be systematically missing from denominators for data abstraction simplification; for example, HIV testing uptake denominators include only those children and adolescents accessing care at outpatient and inpatient facilities and would not include those seeking other services (e.g. family planning, specialty clinics). Further details are described elsewhere [7].

### Statistical analysis

We considered the baseline period to be the six months prior to facility training in SAIA-PEDS (July 2017–December 2017); we considered the implementation strategy period to be the 6 months following the facility training in SAIA-PEDS (January 2018–June 2018). We conducted a simple pre-post analysis and interrupted time series analyses using linear mixed effects models, including random intercepts and random slopes to account for health facility clustering. Model parameterization details are included in the Appendix. We conducted five separate models for each of the five study outcomes. The presented average monthly counts are modeled values that are geometric means across 5 facilities derived from linear mixed-effects models utilizing log transformed values. Changes were considered substantial if they were 20% greater or 20% less than the null value (relative risks of ≥ 1.2 or ≤ 0.8). All analyses were conducted using STATA 14 (StataCorp. 2019. Stata Statistical Software: Release 16. College Station, TX: StataCorp LLC), and all plots were created using R (R Core Team, 2013).

## Results

### Baseline indicators

Among the five facilities included in this evaluation analysis, baseline values of the five outcomes were heterogeneous over 6 months both in their numerator and denominator count data, as well as the ratio of the numerator to denominator. Particularly high monthly indicators often coincided with outreach activities or special focus initiatives.

Based on the pre-post analysis, per facility, the monthly average number of children/adolescents attending outpatient and inpatient services eligible for HIV testing was 842; on average, 253 received HIV testing services, 6 tested positive, 6 were linked to care, and 5 initiated ART. Among those on treatment at the facility, an average of 15 had a VL sample taken and 13 had suppressed VL results returned.

Based on the interrupted time series analysis, the overall baseline temporal trend in the ratio of each indicator among the five facilities was neither significantly increasing or nor decreasing (HIV testing ratio RR: 0.998 [95% CI: 0.860, 1.158]; linkage to care ratio RR: 1.04 [95% CI: 0.880, 1.235]; ART initiation ratio RR: 0.990 [95% CI: 0.854, 1.148]; VL ordering ratio RR: 1.074 [95% CI: 0.908, 1.271]; VL suppression ratio RR: 1.005 [95% CI: 0.892, 1.132]) (Table 1). Due to the negligible baseline temporal trends in the interrupted time series analysis, we present the simple pre-post as the primary results and ITS as secondary model results.

**Table 1:** Regression analyses using pre-post model and interrupted time series

		Baseline average monthly volumes across 5 clinics^	Pre-post model		Interrupted time series model					
					Baseline temporal trend		Step change		Slope change	
			RR	95% CI	RR	95% CI	RR	95% CI	RR	95% CI
Uptake of HIV testing	Numerator	253	1.449	[0.627, 3.347]	1.118	[0.959, 1.305]	1.154	[0.731, 1.823]	0.846**	[0.750, 0.955]
	Denominator	842	1.819***	[1.346, 2.457]	1.121**	[1.038, 1.210]	1.077	[0.759, 1.527]	0.932	[0.849, 1.023]
	Ratio	--	0.803	[0.420, 1.532]	0.998	[0.860, 1.158]	1.079	[0.645, 1.807]	0.905	[0.788, 1.039]
Linkage to HIV care	Numerator	6	0.841	[0.621, 1.139]	0.973	[0.859, 1.102]	0.890	[0.485, 1.633]	1.044	[0.882, 1.236]
	Denominator	6	1.055	[0.659, 1.691]	0.933	[0.809, 1.076]	1.589	[0.849, 2.974]	1.013	[0.859, 1.196]
	Ratio	--	0.831	[0.546, 1.266]	1.042	[0.880, 1.235]	0.549	[0.246, 1.224]	1.057	[0.844, 1.325]
ART initiation	Numerator	5	1.264	[0.850, 1.880]	0.992	[0.852, 1.156]	1.302	[0.668, 2.540]	0.981	[0.818, 1.176]
	Denominator	6	0.841	[0.621, 1.139]	0.973	[0.859, 1.102]	0.890	[0.485, 1.633]	1.044	[0.882, 1.236]
	Ratio	--	1.412	[0.999, 1.996]	0.990	[0.854, 1.148]	1.556	[0.773, 3.131]	0.987	[0.814, 1.198]
VL order	Numerator	15	1.896***	[1.366, 2.633]	1.093	[0.949, 1.259]	1.792	[0.940, 3.419]	0.845	[0.709, 1.007]
	*Excluding extreme clinic*	20	1.469*	[1.073, 2.010]	1.170*	[1.035, 1.323]	1.291	[0.743, 2.244]	0.752***	[0.646, 0.876]
	Ratio	--	1.939**	[1.230, 3.055]	1.074	[0.908, 1.271]	1.810	[0.850, 3.855]	0.886	[0.720, 1.090]
	*Excluding extreme clinic*	--	1.408	[0.981, 2.021]	1.171*	[1.010, 1.358]	1.168	[0.613, 2.229]	0.770**	[0.643, 0.923]
VL suppressed	Numerator	13	1.595**	[1.189, 2.140]	1.099	[0.967, 1.249]	1.214	[0.676, 2.180]	0.899	[0.766, 1.054]
	*Excluding extreme clinic*	13	1.642**	[1.174, 2.298]	1.092	[0.942, 1.265]	1.379	[0.709, 2.682]	0.883	[0.736, 1.060]
	Denominator	15	1.896***	[1.366, 2.633]	1.093	[0.949, 1.259]	1.792	[0.940, 3.419]	0.845	[0.709, 1.007]
	*Excluding extreme clinic*	20	1.469*	[1.073, 2.010]	1.170*	[1.035, 1.323]	1.291	[0.743, 2.244]	0.752***	[0.646, 0.876]
	Ratio	--	0.851	[0.554, 1.306]	1.005	[0.892, 1.132]	0.677	[0.392, 1.169]	1.064	[0.917, 1.234]
	*Excluding extreme clinic*	--	1.119	[0.880, 1.422]	0.934	[0.846, 1.032]	1.065	[0.678, 1.674]	1.172*	[1.036, 1.325]

*p < 0.05, **p < 0.01, ***p < 0.001

^Modeled values represent geometric means across 5 facilities derived from linear mixed-effects models utilizing log transformed values

### Change concepts tested

During the implementation strategy period, a total of 17 change concepts were tested between the five facilities, ranging between two and four changes tested per facility (Table 2). There were eight changes focused on flow reorganization, three focused on newly utilizing checklists or registers, and one each focused on patient navigation, visual cues for providers, job aids for providers, and expanded hours of operation; two had insufficient details to be categorized. Flow reorganization changes focused on addressing waiting time barriers and unclear patient pathways; utilizing checklists, registers, visual cues, and job aids for providers addressed barriers to inconsistent care provision; patient navigation addressed unclear patient flows within complex systems; expanded hours of operation addressed incompatibility between patient availability and service provision times. The majority (nine) of change concepts focused on HIV testing and counseling services, with three focused on linkage to care, three focused on HIV care and treatment, and one focused on VL monitoring; one had insufficient details to be categorized (Table 2). There was moderate alignment between the steps targeted through change concepts and the steps identified as the largest gaps in the facility PedCATs. Across the 6 intervention months at the 5 facilities, the steps most commonly identified as high priority for improvement were as follows: HIV testing (13 occurrences), VL suppression (12 occurrences), VL ordering (8 occurrences), ART initiation (one occurrence), and linkage to care (1 occurrence). Among the 17 change concepts tested, 14 were adopted for further use and three were abandoned.

**Table 2:** Change concepts tested at each facility

Facility number	Type of change	Cascade step focused on
**1**	**Flow reorganization** to reduce missed opportunities for HIV screening	HIV treatment (ART)
	**Flow reorganization** to fast track urgent cases	HIV testing
	**Flow reorganization** to co-locate service delivery for patient ease	HIV treatment (ART)
	**Checklist** introduced to focus on specific patients	VL monitoring
**2**	(unclear)	Linkage to care
	**Patient navigator**/escort	Linkage to care
**3**	**Flow reorganization** to reduce missed opportunities for HIV testing	HIV testing
	**Checklist**/register introduced to expand reach of screening	HIV testing
	**Visual cue** to prompt service provision and using **data** from multiple sources to diagnose drop off daily	HIV testing
	(unclear)	HIV testing
**4**	**Checklist**/register introduced to expand reach of screening	HIV testing
	**Flow reorganization** to reduce missed opportunities for HIV screening	HIV testing
	**Flow reorganization** to reduce missed opportunities for HIV screening	HIV testing
**5**	**Flow reorganization** to fast track urgent cases	Linkage to care
	**Flow reorganization** to reduce missed opportunities for ART adherence	HIV treatment (ART)
	**Expand hours** of operation	All
	Created new **job aid**	HIV testing

### Change associated with strategy

**Fig. 2:**
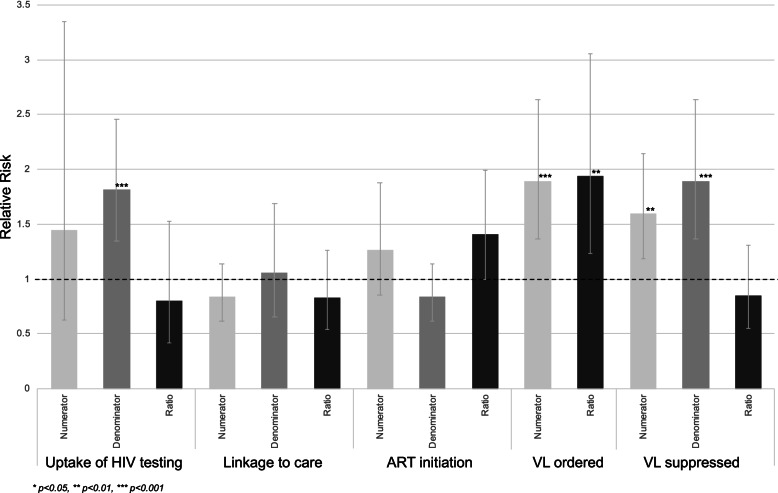
Pre-post plots of point estimates (bars) and 95% confidence intervals (gray whiskers) of change in indicators (numerator, denominator, and ratio) for children and adolescents ages 0–24 years. Dotted black line shows null value of relative risk of 1.0

Using a pre-post design comparing the 6 months prior to the implementation strategy to the 6 months of the implementation strategy period, there was no substantial or significant change in the ratio of HIV testing (RR: 0.803 [95% CI: 0.420, 1.532]) and linkage to care (RR: 0.831 [95% CI: 0.546, 1.266]). The ratio of ART initiation increased substantially and trended towards significance (RR: 1.412 [95% CI: 0.999, 1.996]). There were significant and substantial improvements in the ratio of VL tests ordered (RR: 1.939 [95% CI: 1.230, 3.055]) but no substantial or significant change in the ratio of VL results suppressed (RR: 0.851 [95% CI: 0.554, 1.306]) (Table 1; Fig. 2).

**Fig. 3:**
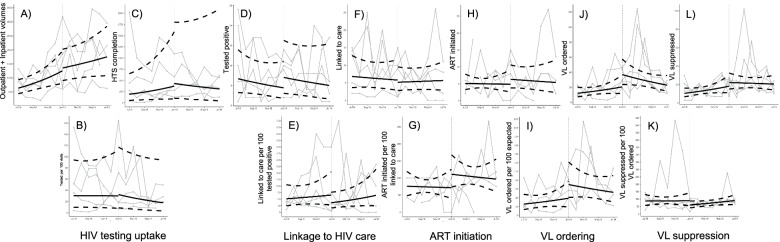
Interrupted time series plots of counts and ratios of indicators for children and adolescents ages 0–24 years; facility specific data in gray solid lines; fitted model result point estimates (in solid black line) and 95% confidence intervals (dotted black line); implementation strategy started at vertical dotted line in January 2019. **A** Number of outpatient and inpatient clients, **B** ratio of HIV testing uptake: eligible outpatient and inpatient visits (ineligible removed from denominator), **C** number with HIV testing services (HTS) completed, **D** number testing HIV positive, **E** ratio of clients linked to care: those testing positive, **F** number linked to care, **G** ratio of clients initiating antiretroviral therapy (ART) same day among those linked to care, **H** number initiating ART, **I** ratio of clients with viral load (VL) ordered: those with VL due (fixed value per month), **J** number with VL ordered, **K** ratio of clients with suppressed VL: those with VL ordered, **L** number with suppressed VL

Despite no change in the ratio of HIV testing uptake, both the numerator (those who completed HIV testing) and denominator (those who presented to in- and out-patient clinics and were eligible for HIV testing) both increased substantially during the implementation strategy period, a change that was significant in the denominator only (HIV testing RR: 1.449 [95% CI: 0.627, 3.347]; in- and out-patient clients eligible RR: 1.819 [95% CI: 1.346, 2.457]). In contrast, there was a relatively small change in the numerator and no change in the denominator for linkage to care (RR: 0.841 [95% CI: 0.621, 1.139]; RR: 1.055 [95% CI: 0.659, 1.691], respectively). The substantial but not significant change in the ratio of ART initiation was driven by the increase in the numerator of those who initiated ART (RR: 1.264 [95% CI: 0.850, 1.880]) and the relatively small decrease in those testing positive (RR: 0.841 [95% CI: 0.621, 1.139]) (Table 1; Fig. 3).

The VL ordering and VL suppression outcomes had one facility with particularly high values. When that one facility was removed, the magnitude of the change in the ratio of VL ordered was reduced and only trended towards statistical significance (ratio VL ordered RR: 1.408 [95% CI: 0.981, 2.021]), driven by the increases in the numerator paired with a constant denominator. When that one facility was removed, the magnitude of the change in the ratio of VL suppressed was relatively unchanged. However, both the numerator (VL suppressed) and denominator (VL ordered) increased substantially and significantly, improvements which were retained when the one high value facility was removed (Table 1). In site specific sub-analyses, facility 1 (the only facility that tested a change concept focused on VL monitoring) drove the observed association, having the largest or second largest effect sizes in VL suppression numerator and denominator.

In the interrupted time series analysis, there were no significant improvements or reductions in the step change at the time of the introduction of the implementation strategy, and there were no clear messages in the improvements or reductions in the slope change during the implementation strategy period (Table 1; Fig. 2).

## Discussion

In this five-facility pilot study of an adapted version of the SAIA multi-component implementation strategy, we assessed the impact of the implementation strategy on pediatric and adolescent HIV testing and treatment cascade indicators. We observed heterogeneous results: during the implementation strategy period, we observed substantial and significant increases in the number of individuals seeking inpatient and outpatient services, the number of viral load samples ordered, the number of viral loads that were suppressed, and the ratio of viral loads ordered compared to viral loads due. The implementation strategy was associated with substantial, but only trending towards significant, improvements in the ratio of those who initiated ART compared to those testing HIV positive. The implementation strategy was not associated with substantial or significant improvements in HIV testing or linkage to care.

The ratio of HIV testing compared to those seeking care and the numbers and ratios of those linking to care were not observed to improve with the implementation strategy, with slight and non-significant decreases in the ratios of testing and linkage to care. This was surprising, given the large number of change concepts that focused on this step of the cascade. There was strong heterogeneity between facilities in the eligibility assessments for HIV testing services; some facilities routinely screened for eligibility, while others did not, and some used a Ministry of Health register with certain criteria while others used implementing partner registries with different criteria. One facility tested a change concept of using a checklist to begin eligibility screening during the implementation strategy period. Importantly, a large health care worker strike resolved at the same time that the implementation strategy was introduced; as such, we observed a substantial and significant increase in the number of individuals seeking inpatient and outpatient services; this was paired with an increase that did not quite keep pace in HIV testing completion. It is likely that the implementation strategy resulted in an increase in HIV testing coverage, but that change was overshadowed proportionally by the massive increase in demand for services overall after the strike resolution. Given the data collection simplifications that were necessary to make the PedCAT tool feasible, including only abstracting the counts of individuals seeking inpatient and outpatient services and not specialty clinics or other entry points within a health center, it is not possible to calculate a true estimate of HIV testing coverage. Had this been feasible, we could have assessed whether this indicator began with high coverage and did not have opportunity to increase, as was noted in the original SAIA trial [8]. Other studies have noted that quality improvement has increased HIV testing coverage substantially [24]; it is unlikely that this implementation strategy would be detrimental to these services.

Linkage to care was particularly challenging to assess and heterogeneous between facilities due to issues of migration, lagged windows for linkage, and duplicate data sources. Individuals living with HIV may prefer to link to HIV care at a different clinic due to stigma [25]; this in and out migration of individuals is both appropriate to meet patient care needs and complicated for data systems due to lack of nation-wide unique identifier systems. Facilities with substantially more in-migration may artificially appear to be performing better than those facilities with more out migration. Unlike HIV testing, which is assessed same-day cross-sectionally, linkage to care is often operationalized with a 1-month window (as was done in this study), meaning that individuals may be diagnosed with HIV and link to care within separate month windows for data aggregation. Finally, linkage to care is often the first time when health facilities that use HIV care electronic medical records enter a patient into their databases. One facility in our study captured linkage to care numbers both in their electronic medical records system as well as paper registers, sources which were inconsistent with one another. From a pragmatic standpoint, the numerator for this indicator was one of the most challenging to abstract from routine program data sources due to the manual assessment of the 1-month diagnosis to linkage window and multiple data sources.

ART initiation within 1 month of linkage to care was substantially, but not significantly, higher in the implementation strategy period than the baseline period. There were three change concepts tested that focused on this cascade step (Table 2). Our findings were similar to the original SAIA trial, in which the implementation strategy was associated with a three-fold non-significant improvement in ART initiation for mothers [8]. While not significant, this is promising for a pilot study effect, given the massive impact that prompt ART has on child mortality and morbidity reduction when given prior to symptomatic disease [26–28].

The positive association of the implementation strategy with the number of VL ordered, the ratio of VL ordered compared to VL due, and the number of VL samples suppressed was unexpected. VL suppression is affected by many factors, particularly those at the individual and interpersonal levels, and it was not expected to respond to a health systems implementation strategy strongly. Additionally, just one change concept was focused specifically on VL testing within this pilot. The number of VL tests due was calculated based on national guidelines rather than being abstracted from records due to the massive complexity in direct assessment; this fixed number did not vary monthly, an assumption which was in line with lack of predicted seasonality in HIV care visits, but may not have accounted for natural heterogeneity in visit schedules. Finally, the relatively long turnaround time for VL samples in the Kenyan centralized laboratory system [29] meant that individuals likely had a VL sample collected and results returned in separate month windows for data aggregation.

This pilot study had numerous strengths. The study indicators aligned well with the UNAIDS 95-95-95 goals, which were also Kenyan national guidelines. It used a quasi-experimental analysis method to assess whether baseline temporal trends were driving the magnitude of implementation strategy impact, it included facilities in diverse regions in Kenya, and it utilized routine program data sources without any primary data collection. It began with an effective multi-component implementation strategy and adapted it in partnership with stakeholders to be relevant and applicable to the pediatric and adolescent cascade. A full qualitative evaluation of the implementation strategy was conducted and presented elsewhere (*Wagner & Beima-Sofie, under review*).

This study also had several limitations. A pre-post analysis is a relatively weak design for determining impact; even an uncontrolled interrupted time series analysis is vulnerable to external temporal changes. In this study, the implementation strategy began at the same time that health service provision resumed after a multi-month strike, which seriously weakens the inference about the independent impact of the implementation strategy. However, we assessed changes in the numerator and denominator of indicators, which partially accounts for changes observed both in demand for services and supply of services separately. Secondly, the PedCAT tool was not available at all facilities for the first few months of implementation due to delays in data abstraction, which limited the use of this prioritization tool. Thirdly, it was challenging to maintain fidelity to the intended implementation strategy coaching visit schedule due to competing service provision priorities, potentially impactive the “dose” of the implementation strategy delivered. Fourthly, lack of financial reimbursement to facility providers negatively impacted willingness to participate in CQI meetings. Future assessments of this implementation strategy would need to carefully address fidelity and select an evaluation design that is robust to temporal changes. Fifthly, the implementation strategy period of 6 months was relatively short and there were relatively few change concepts tested (compared to the original trial), potentially insufficient time and number of change concepts to observe implementation strategy impact on some indicators. Finally, the abstracted data could not accurately be interpreted as proportions or coverage due to incomplete denominators and in and out migration, limiting the ease of interpretation and alignment with set indicators like the UNAIDS 95-95-95 goals.

## Conclusions

In conclusion, we saw that the adapted SAIA-PEDS implementation strategy was associated with significant and substantial improvement in some pediatric and adolescent HIV cascade indicators, including viral load monitoring and suppression, and trended towards significant impact on ART initiation. Given the critical and urgent nature of pediatric and adolescent HIV testing and treatment, the relative flexibility of this implementation strategy to meet local contexts’ needs and structures, and demonstrated impact in other settings, this pilot merits follow-up with a cluster randomized trial for rigorous evaluation in diverse contexts.

## Data Availability

The datasets used during the current study are available from the corresponding author on reasonable request.

## Appendix

### Modeling equations

#### Pre/Post

$$\log \left[{\left( count\ or\ ratio\right)}_{ht}\right]=\left({\alpha}_0+{b}_{0h}\right)+\left({\alpha}_1+{b}_{1h}\right)\cdot post$$

where h indexes health facilities and t indexes time in months.

*count or ratio* is the outcome as an aggregated count or ratio of two counts.

*post* is 1 for the last 6 months (the implementation strategy period).

*b*_0*h*_ and *b*_1*h*_ are random intercepts and slopes, respectively.

Therefore, ${e}^{\alpha_{1}}$ is the step change trend. A relative change of the average counts or ratios from the first 6 months to the 6 months of implementation strategy

#### Interrupted time series analysis

$${\displaystyle \begin{array}{r}\log \left[{\left( count\ or\ ratio\right)}_{ht}\right]=\left({\beta}_0+{b}_{0h}\right)+\left({\beta}_1+{b}_{1h}\right)\cdot time+{\beta}_2\cdot post+{\beta}_3\cdot time post\end{array}}$$

where *h* indexes health facilities and t indexes time in months.

*count or ratio* is the outcome as an aggregated count or ratio of two counts.

*time* counts the months from 0 to 11.

*post* is 1 for the last 6 months (the implementation strategy period).

*timepost* counts time since the 7^*th*^ month. Before that is zero.

*β*_0_ is the average log-counts (or ratio) at the beginning of the study period.

*β*_1_ is the 6 months pre-implementation strategy slope.

*β*_2_ is the immediate level change at beginning of the implementation strategy period.

*β*_3_ is the change in *β*_1_ slope.

*b*_0*h*_ and *b*_1*h*_ are random intercepts and slopes, respectively.

Therefore, ${e}^{\beta_1}$ is the baseline monthly relative trend; ${e}^{\beta_2}$ is the step change trend; ${e}^{\beta_3}$ is relative change of the baseline trend.
